# In vitro cytotoxicity of self-curing acrylic resins of different colors

**DOI:** 10.1590/2176-9451.19.4.066-070.oar

**Published:** 2014

**Authors:** Luciana Borges Retamoso, Taís de Morais Alves da Cunha, Matheus Melo Pithon, Rogério Lacerda dos Santos, Fernanda Otaviano Martins, Maria Teresa Villela Romanos, Orlando Motohiro Tanaka

**Affiliations:** 1 PhD resident in Dental Material, Catholic University of Rio Grande do Sul (PUC-RS).; 2 MSc in Orthodontics, Catholic University of Paraná (PUC-PR).; 3 PhD resident in Orthodontics, Federal University of Rio de Janeiro (UFRJ).; 4 MSc in Immunobiological Technology, Oswaldo Cruz Foundation (FIOCRUZ).; 5 Adjunct Professor, UFRJ.; 6 Full Professor, Department of Orthodontics, –PUCPR.

**Keywords:** Acrylic resins, Cell culture techniques, Cytotoxins

## Abstract

**Objective:**

The aim of this study was to assess the *in vitro* cytotoxicity of acrylic resins of different colors over time.

**Methods:**

Specimens were divided into 4 groups (n = 6) according to the color of the acrylic resin (Orto Class, Clássico, Campinas, São Paulo, Brazil): Group 1: clear acrylic resin; group 2: pink acrylic resin; group 3: blue acrylic resin and group 4: green acrylic resin. All specimens were fabricated according to the mass manipulation technique and submitted to mechanical polishing protocol. The control was performed with an amalgam specimen (C+), a glass specimen (C-) and cell control (CC). Specimens were immersed in Minimum Eagle's Medium (MEM) and incubated for 24 h at 37ºC. The extracts from the experimental material were filtered and mixed with L929 fibroblast. Cytotoxicity was evaluated at 4 different times, 24, 48, 72 and 168 h. After contact, cells were incubated for 24 h and added to 100 µ of 0.01% neutral red dye. The cells were incubated for 3 h for pigment incorporation and fixed. Cells viability was determined by a spectroscopic (BioTek, Winooski, Vermont, USA) with a 492-nm wavelength λ=492 nm).

**Results:**

There were no statistical differences between the experimental groups and the CC and C- groups.

**Conclusion:**

Clear, pink, blue and green self-curing acrylic resins fabricated by means of the mass manipulation technique and mechanically polished are not cytotoxic. Neither the pigment added to the self-curing acrylic resin nor the factor of time influenced the cytotoxicity of the material.

## Abstract (pt)

**Objetivo:**

avaliar, *in vitro*, a citotoxicidade de resinas acrílicas autopolimerizáveis, de diferentes cores, ao longo do tempo.

**Métodos:**

os corpos de prova foram divididos em quatro grupos (n = 3), de acordo com a cor da resina acrílica utilizada (Orto Class, Clássico, São Paulo/SP), sendo: grupo 1, acrílica incolor; grupo 2, acrílica rosa; grupo 3, acrílica azul; e, grupo 4, acrílico verde. Todos os corpos de prova foram confeccionados pela técnica de massa e polidos mecanicamente. Um corpo de prova de amálgama, um de vidro e célula constituíram o controle positivo (C+), controle negativo (C-), e controle de célula (CC), respectivamente. Em seguida, esses foram imersos em meio mínimo essencial de Eagle (MEM) por 24h, quando se removeu o sobrenadante e colocou-os em contato com fibroblastos L929. Avaliou-se a citotoxicidade em quatro períodos: 24, 48, 72 e 168h. Após o contato com o meio, as células foram incubadas por 24h e adicionou-se 100µ do corante vermelho neutro a 0,01%. Posteriormente, as células foram incubadas por 3h, para incorporação do corante, e fixadas. A contagem das células viáveis foi realizada em espectrofotômetro (BioTek, Winooski, EUA), com um comprimento de onda de 492nm (λ = 492nm).

**Resultados:**

não houve diferença estatística entre os grupos experimentais e os grupos CC e C-.

**Conclusções:**

as resinas acrílicas autopolimerizáveis incolor, rosa, azul e verde, manipuladas pela técnica de massa e polidas mecanicamente não são citotóxicas. O corante utilizado em resinas autopolimerizáveis e tempo não influenciam na citotoxocidade do material.

## INTRODUCTION

Chemically activated acrylic resins are widely used in the fabrication of fixed, removable and retention orthodontic appliances.^4^ Resin is sold in two vials: one containing the powder, the polymer, and the other containing the liquid, the monomer. The monomer is a clear, flammable and volatile liquid at room temperature.^1^ It is considered cytotoxic and possibly genotoxic.^9,10,22,23,24^ The polymer usually has the pigment that gives color to the resin.

Adding the monomer (methyl methacrylate, MMA) to the polymer causes a resin polymerization reaction that occurs without the formation of byproducts. Nevertheless, conversion of monomer into polymer is generally not complete,^4^ for this reason, some amount of monomer, known as residual,^2^ remains. According to some studies, residual monomer remains in the manufactured orthodontic appliances, which indicates that varying amounts of residual monomer may be released into the oral cavity during the use of these appliances.^4,10^

The residual monomer of methyl methacrylate not only changes the final physical properties of resins,^3,12^ but also induces the onset of systemic and local tissue reactions when in contact with saliva and soft tissues,^8,9,11,17^ thus causing hypersensitivity, lip swelling, chronic urticaria and sialorrhea.^5,6^ Furthermore, the pigment added to the powder may be another causative factor of hypersensitivity.

Although the *in vitro* cytotoxicity of MMA has already been demonstrated, no studies were conducted to assess the influence of pigments present in colored acrylics on cell viability. Thus, the aim of this study was to evaluate the *in vitro *cytotoxicity of acrylic resin at different periods and compare the cytotoxicity of acrylic resins of different colors.

## MATERIAL AND METHODS

### Preparation of specimens

For preparation of specimens, a metal matrix (10 mm X 5 mm X 2 mm) was molded with addition silicone (Express^®^, 3M/ESPE, St. Paul, USA) and the mold filled with self-curing acrylic resin (Ortho Class^®^, Classic, Campinas, São Paulo, Brazil). Powder-liquid ratio was obtained according to the manufacturer's instructions.

Each acrylic resin used for preparation of specimens was manipulated by means of the mass technique in a dappen dish with a lid where the monomer was inserted immediately before the polymer was poured until its saturation. Subsequently, the dish was covered with the lid, which allowed the resin to go through a sandy and fibrillar phase until it reached its plastic phase during which it was inserted into the mold. The acrylic resin was processed in a resin polymerizer M 1000^®^ (EDG Equipment and Control Ltda.) at 20°C and pressure of 25 psi (1.75 kg/cm^2^) for a period of 15 minutes.

After polymerization, mechanical polishing was carried out in a vise using a bristle brush with a mixture pumice and water for 1 minute, followed by felt with white paste of Spain used for 1 minute.

### Groups

Four self-curing acrylic resins of different colors were divided into four groups as follows (n = 3): Group 1 (clear), 2 (pink), 3 (blue) and 4 (green).

### Control

To assess cellular response against extremes, other three groups (n = 3) were included: Group CC (cell control), cells which were not exposed to any material. This group was used to monitor normal cell growth. Group C+ (positive control) consisting of specimen made ​of amalgam. Silver amalgam was used because of its well known cytotoxic ability.^18^ Specimens of 10 mm x 5 mm x 2 mm were manufactured in amalgamator (SDI^®^, Bayswater, Australia) and polished with abrasive rubber tips. Group C- (negative control) consisting of glass specimen. Glass was the material of choice for not triggering cytotoxicity effect.^19^

### Cell culture

Cell lineage used was L929 obtained from the American Type Culture Collection (ATCC, Rockville, MD) (mouse fibroblasts) grown in Eagle's minimum essential medium (MEM) (Cultilab, Campinas, São Paulo, Brazil), supplemented with 2 mM L glutamine (Sigma, St. Louis, Missouri, USA) 50 µg/mL gentamycin (Schering Plough, Kenilworth, New Jersey, USA), 2.5 µg/mL fungizone (Bristol Myers Squibb, New York, USA), 0.25 mL sodium bicarbonate solution (Merck, Darmstadt, Germany), 10 mM HEPES (Sigma, St. Louis, Missouri, USA) and 10 % fetal bovine serum (FBS) (Cultilab, Campinas, São Paulo, Brazil). It was kept at 37ºC in an environment supplemented with 5 % CO_2_.

### Cytotoxicity assay

Acrylic resin, silver amalgam and glass specimens were sterilized by exposure to UV light (Labconco, Kansas, Missouri, USA) for 1 hour.^19^ Then, three samples of each material were placed in 24 well plates containing culture medium (MEM) (Cultilab, Campinas, São Paulo, Brazil). Supernatants were collected according to the time of evaluation, 24, 48, 72 and 168 hours (7 days), being the culture medium renewed every 24 hours.

Supernatants were placed, in triplicate, in 96 well plates containing confluent monolayer of L929 cells and incubated for 24 hours at 37ºC in an environment containing 5 % CO_2_. After incubation, the effect on cell viability was determined by means of the dye-uptake technique, as described by Neyndorff et al,^16^ but with minor modifications. The technique consists in adding 100 µL of 0.01 % neutral red (Sigma, St. Louis, Missouri, USA) into culture medium and incubation at 37°C for 3 hours for penetration of the dye in living cells.

After this period, the dye was discarded and the cells fixed for 5 minutes by adding 100 µL of formaldehyde solution (Reagen, Rio de Janeiro, Rio de Janeiro, Brazil) to 4 % in PBS (130 mM NaCl, 2 mM KCl; 6 mM Na_2_HPO_4_.2H_2_O, 1 mM K_2_HPO_4_, pH 7.2). Subsequently, the dye was extracted by adding 100 µL of a solution of 1 % acetic acid (Vetec, Rio de Janeiro, Rio de Janeiro, Brazil) and 50 % methanol (Reagen, Rio de Janeiro, Rio de Janeiro, Brazil). After 20 minutes, a spectrophotometer (BioTek, Winooski, Vermont, USA) at a wavelength of 492 ηm (λ = 492 ηm) was used to read the data.

### Statistical analysis

Statistical analysis was performed with the SPSS 13.0 software (SPSS Inc., Chicago, Illinois, USA). Initially, data were submitted to Kolmogorov Smirnov and Levene's test to determine normality and homogeneity, respectively. The values ​​of the amount of viable cells were subjected to analysis of variance (ANOVA), with two factors (color and time) to determine whether there were statistical differences between groups, and subsequently to Tukey's test (Table 1). Significance level was set at 5 %.

**Table 1:** Mean and standard deviation of the amount of viable cells and statistical analysis of evaluated groups.

	24 h		48 h		72 h		168 h	
Groups	Mean ± SD	St.	Mean ± SD	St.	Mean ± SD	St.	Mean ± SD	St.
1	690 ± 139.7	^AC^	1061 ± 76.25	^A^	854.3 ± 65.4	^A^	1213.1 ± 190.3	^A^
2	584.6 ± 81.1	^A^	1099.3 ± 116.4	^A^	876.6 ± 59.6	^A^	1281.1 ± 92.89	^A^
3	679.6 ± 117.8	^AC^	1137.2 ± 70.74	^A^	828.6 ± 134.3	^A^	1147.7 ± 91.53	^A^
4	583.3 ± 57.8	^A^	1143.2 ± 102.95	^A^	869.6 ± 72.36	^A^	1199.3 ± 102.3	^A^
C+	324.2 ± 25.4	^B^	550.6 ± 91.87	^B^	404 ± 59.4	^B^	580.7 ± 46.3	^B^
C-	708.4 ± 64.0	^AC^	1068.7 ± 178.7	^A^	918.6 ± 34.0	^A^	1228.2 ± 137.2	^A^
CC	730.6 ± 84.8	^C^	1108.4 ± 45.94	^A^	942.6 ± 49.14	^A^	1292.8 ± 143.9	^A^

Mean: mean values of the amount of viable cells;

SD: Standard deviation;

St: Statistics. Same letters account for the absence of statistical difference for Tukey's test (p < 0.05).

## RESULTS

Results revealed increased cell viability from 24 to 48 h, a reduction in cell viability after 72 h and, an increase in cell viability after 168 h; however, with no statistically significant differences (P ≥ 0.05).

The color of resin proved not to influence material cytotoxicity, since there were no differences between groups 1, 2, 3 and 4 at all times (P ≥ 0.05).

At all times, the values ​​of cell viability were statistically similar between clear acrylic resin (group 1) and blue acrylic resin (group 3), as well as between groups C- and CC (P ≥ 0.05), thus proving the absence of cytotoxic effect of acrylic resin to fibroblasts. However, experimental group 2 (pink acrylic resin) and 4 (green acrylic resin) showed significant differences in comparison to group CC (P < 0.05) after 24 h, thus indicating a difference in normal cell growth. Group C+ showed statistical difference in comparison to all other groups at all time periods studied, thus showing a decrease in the number of viable cells.

## DISCUSSION

Acrylic resins are widely used in Dentistry; however, some studies have demonstrated that this material can cause allergic reactions.^13,14,15^ Nevertheless, most researches assessed material used for prosthetic purposes, most of which are heat-curing

Self-curing ​​acrylic resins are the most frequently used in Orthodontics. According to Hensten Pettersen and Wictorin,^7^ polymerization influences cytotoxicity. Their studies revealed lower cell growth in self-curing ​​resins in comparison to heat-curing ones, and for both, cell growth was lower than in the control group.

Baker et al^2^ found that residual monomer concentration was four times higher in saliva adjacent to the palatal surface of appliances manufactured ​​with acrylic resin in comparison to total saliva, thus indicating the importance of assessing cytotoxicity of this material, as well as the effects produced by the pigment and by the time of exposure on material cytotoxic potential.

Acrylic resin color proved not to affect cell viability, thus suggesting that the pigment does not influence cytotoxicity levels. However, when specimens were made ​​of pink and green acrylic resin, normal cell growth was modified, as shown by experimental groups 2 and 4 which differed from CC. Thus, toxic reaction seems not to be associated with neither pigment nor the other substances that constitute the polymer, but with increase in the level of residual monomer present in the material.^6^

In this study, groups 1, 2, 3 and 4 showed no statistically significant differences over time (P ≤ 0.05), thus indicating that cytotoxicity was not affected within the times tested. This finding is in disagreement with Gonçalves et al^6^ who assessed cytotoxicity of acrylic resins for orthodontic purposes within 24 and 48 hours. Their results showed that there was less cell viability after 24 hours. This difference can be explained by the cell type used, given that Gonçalves et al^6^ used epithelial cells and not fibroblasts. Polishing may also be considered as, according to Rocha Filho et al^20^ and Gonçalves et al,^4^ it alters the level of residual monomer present in the acrylic resins. Release of residual monomer is responsible for the reduction in cell viability, and this release is more intense within the first 24 h.^4^ However, mechanical polishing decreases the levels of residual monomer^6^. For this reason, it is suggested that the polishing procedure performed in this study was key not to trigger toxic reaction.

Although there was no significant difference between the times of assessment, in all experimental groups, from 48 to 72 hours there was a decrease in the number of viable cells. The fact that release of residual monomer is considered crucial in determining cell viability corroborates the results by Rocha Filho et al.^20^ In the graphs of this study, the authors demonstrate increased concentration of residual monomer from 2 to 5 days.

It is recommended that acrylic resin used to manufacture orthodontic appliances be properly proportioned and manipulated, following the manufacturer's recommendations to ensure safety for patients' health. Some measures may be taken to reduce the amount of residual monomer, such as: polymerization in water or under pressure, the use of correct monomer : polymer proportion, and storage in water for 72 hours after polymerization.^2,17,21^ Moreover, the different colors of resin tested can be used without causing any damage to the biocompatibility of the material.

## CONCLUSION

According to the methodology used and the conditions established in this research, it can be concluded that:

1. Acrylic resin manufactured by means of the mass technique, polymerized under pressure and mechanically polished does not alter cell viability.
2. Color of acrylic resin has no effect on cell viability.
3. Cell viability is maintained when exposed to self-curing acrylic resin.
